# Oviposition ecology and species composition of *Aedes* spp. and *Aedes aegypti* dynamics in variously urbanized settings in arbovirus foci in southeastern Côte d’Ivoire

**DOI:** 10.1186/s13071-016-1778-9

**Published:** 2016-09-29

**Authors:** Julien B.Z. Zahouli, Jürg Utzinger, Maurice A. Adja, Pie Müller, David Malone, Yao Tano, Benjamin G. Koudou

**Affiliations:** 1 Unité de Formation et de Recherche Biosciences, Université Félix Houphouët-Boigny, Abidjan, Côte d’Ivoire; 2 Centre Suisse de Recherches Scientifiques en Côte d’Ivoire, Abidjan, Côte d’Ivoire; 3 Swiss Tropical and Public Health Institute, Basel, Switzerland; 4 University of Basel, Basel, Switzerland; 5 Innovative Vector Control Consortium, Liverpool School of Tropical Medicine, Liverpool, UK; 6 Université Nangui-Abrogoua, Abidjan, Côte d’Ivoire; 7 Filariasis Programme Support Unit from Liverpool School of Tropical Medicine, Liverpool, UK

**Keywords:** Arboviruses, *Aedes*, Oviposition ecology, *Culex*, *Eretmapodites*, Ovitrap, Socio-ecological survey, Urbanization, Côte d’Ivoire

## Abstract

**Background:**

*Aedes* mosquito-transmitted outbreaks of dengue and yellow fever have been reported from rural and urban parts of Côte d’Ivoire. The present study aimed at assessing *Aedes* spp. oviposition ecology in variously urbanized settings within arbovirus foci in southeastern Côte d’Ivoire.

**Methods:**

*Aedes* spp*.* eggs were sampled using a standard ovitrap method from January 2013 to April 2014 in different ecosystems of rural, suburban and urban areas. Emerged larvae were reared until the adult stage for species identification.

**Results:**

*Aedes* spp. oviposition ecology significantly varied from rural-to-urban areas and according to the ecozones and the seasons. Species richness of *Aedes* spp. gradually decreased from rural (eight species) to suburban (three species) and urban (one species) areas. Conversely, emerged adult *Aedes* spp. mean numbers were higher in the urban (1.97 *Aedes*/ovitrap/week), followed by the suburban (1.44 *Aedes*/ovitrap/week) and rural (0.89 *Aedes*/ovitrap/week) areas. *Aedes aegypti* was the only species in the urban setting (100 %), and was also the predominant species in suburban (85.5 %) and rural (63.3 %) areas. The highest *Ae. aegypti* mean number was observed in the urban (1.97 *Ae. aegypti*/ovitrap/week), followed by the suburban (1.20 *Ae. aegypti*/ovitrap/week) and rural (0.57 *Ae. aegypti*/ovitrap/week) areas. *Aedes africanus* (9.4 %), *Ae. dendrophilus* (8.0 %), *Ae. metallicus* (1.3 %) in the rural, and *Ae. vittatus* (6.5 %) and *Ae. metallicus* (1.2 %) in the suburban areas each represented more than 1 % of the total *Aedes* fauna. In all areas, *Aedes* species richness and abundance were higher in the peridomestic zones and during the rainy season, with stronger variations in species richness in the rural and in abundance in the urban areas. Besides, the highest *Culex quinquefasciatus* abundance was found in the urban areas, while *Eretmapodites chrysogaster* was restricted to the rural areas.

**Conclusions:**

Urbanization correlates with a substantially higher abundance in *Aedes* mosquitoes and a regression of the *Aedes* wild species towards a unique presence of *Ae. aegypti* in urban areas. *Aedes* wild species serve as bridge vectors of arboviruses in rural areas, while *Ae. aegypti* amplifies arbovirus transmission in urban areas. Our results have important ramifications for dengue and yellow fever vector control and surveillance strategies in arbovirus foci in southeastern Côte d’Ivoire.

**Electronic supplementary material:**

The online version of this article (doi:10.1186/s13071-016-1778-9) contains supplementary material, which is available to authorized users.

## Background

Several *Aedes* mosquito species are involved in the transmission of arboviral diseases, including dengue and yellow fever, responsible for major health burdens worldwide [1–3]. In the mid-1990s, yellow fever was controlled in Francophone Africa by vaccination while both yellow fever and dengue were eliminated in the Americas by effective control of *Aedes aegypti* [4]. However, in recent years, there has been a dramatic resurgence of dengue fever worldwide [5–8] and re-emergence of yellow fever in some parts of Africa [9]. In addition, other arboviruses vectored by *Aedes* mosquitoes, such as chikungunya [10], Rift valley fever [11] and Zika virus [12] are emerging or re-emerging in Africa, particularly in West Africa [13–15]. The patterns of arboviral disease transmission and its geographic expansion are likely a result of intensive urbanization [1, 2, 6, 16]. However, dengue and yellow fever originated in enzootic (sylvatic) cycles associated with wild *Aedes* vectors in rural areas. Enzootic cycles are linked to urban transmission cycles by a major domestic vector, *Ae. aegypti* [17].

In Côte d’Ivoire, single and dual epidemics of dengue and yellow fever involving several wild *Aedes* species and the major urban vector, *Ae. aegypti*, have been reported in both rural and urban areas [18]. Sylvatic dengue virus circulation, without human infections, was documented by isolation of DENV-2 serotype from wild *Aedes* vectors, including *Aedes africanus*, *Aedes furcifer*, *Aedes luteocephalus*, *Aedes opok* and *Aedes vittatus* in rural areas in the 1980s [19]. Since then, several sporadic outbreaks of dengue DENV-1, DENV-2 and DENV-3 serotypes and yellow fever sometimes resulting in fatal outcomes have occurred [20–22]. The outbreaks were mostly concentrated in surrounding villages and periurban areas of Abidjan, the economic capital and the most densely populated city of Côte d’Ivoire [21, 22].

The comprehension of the shaping patterns of immature *Aedes* mosquito ecology along the urbanization gradient is of paramount importance in determining their role in maintenance of epidemic arboviral diseases transmission [1, 2]. Knowledge of such patterns may therefore enable a more effective deployment of vector control measures for the benefit of public health. *Aedes* mosquitoes are readily adapted to a broad range of ecological settings and are expected to vary according to natural and urbanized environment [2, 9]. Certain *Aedes* mosquito species are confined and limited to sylvatic, rural or urban areas, whereas others have a large distribution and colonize almost every environment, such as the wild, rural and urbanized settings, the domestic and peridomestic premises, the types of landscapes and the microhabitats [2, 9]. Those species of *Aedes* occurring in transitional environments may serve as bridge vectors between enzootic diseases and humans in rural areas. Moreover, *Aedes* mosquitoes are the main reservoirs of arboviruses and the longest link of the transmission chain since they host the viruses during longer duration compared to humans and wild animals [23]. These *Aedes* vector species show both oral and transovarial infection [23, 24]. The extent to which eggs are resistant against desiccation varies between species and strains, and depends on climatic conditions [25, 26]. Otherwise, *Aedes* mosquito species can be associated with other mosquito species for different interaction purposes such as predation, competition and symbiosis [9]. *Eretmapodites chrysogaster* is a predaceous mosquito and lays its eggs in *Aedes* species breeding sites [27]. *Aedes* and *Culex* species, mainly *Culex quinquefasciatus* and *Ae. aegypti*, are sympatric and co-occur in the same containers [28].

*Aedes aegypti* is an urban species and a major vector of dengue and yellow fever by amplifying epidemics among the urban populations [9, 17]. This species consists of two subspecies, *Ae. aegypti aegypti* and *Ae. aegypti formosus* that are morphologically [29], behaviourally and genetically distinct [30–32]. However, there are ambiguities resulting in confusion over morphological distinction between the two subspecies of *Ae. aegypti* in West Africa [13, 33].

Urbanization could potentially modify *Aedes* mosquito ecology by changing the composition and dynamics of species, and increasing the abundance of their breeding sites due to environmental changes, and thus contribute to arbovirus outbreaks [2]. However, *Aedes* mosquito egg laying ecology is unknown in arbovirus foci located in variously urbanized settings of southeastern Côte d’Ivoire. To fill this gap, our study explored *Aedes* mosquito egg laying patterns, species composition and dynamics in Ehania-V1, Blockhauss and Treichville representing rural, suburban and urban settings of southeastern Côte d’Ivoire, respectively. Because immature mosquitoes are sensitive to environmental changes [2, 25, 26], we hypothesized that *Aedes* mosquito oviposition ecology and species composition, and the dynamics of *Ae. aegypti* change from rural to suburban and urban settings. Field surveys of *Aedes* mosquito egg were performed using a highly sensitive sampling method, namely the standardized World Health Organization (WHO) ovitraps [23, 24], larval rearing in the laboratory and adult stage identification were conducted to test our hypothesis. The findings provide valuable information on *Aedes* mosquito egg laying patterns, species composition and *Ae. aegypti* dynamics in different urbanized ecosystems. The key results open new perspectives for improving current vector control and surveillance strategies for dengue and yellow fever that are tailored for specific settings of southeastern Côte d’Ivoire.

## Methods

### Study area

**Fig. 1:**
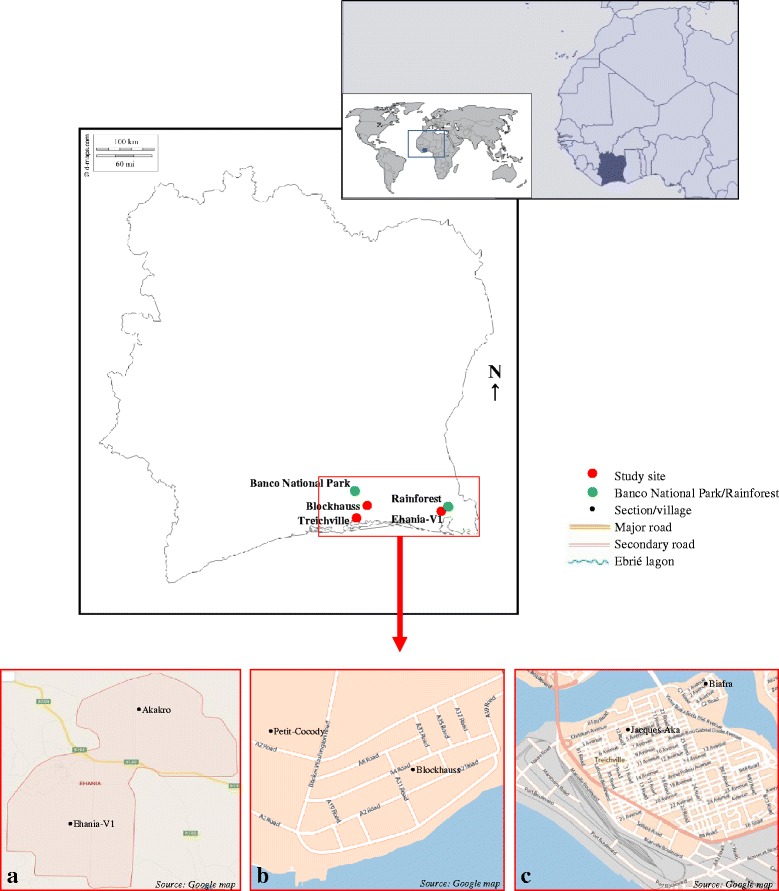
Map of the areas located in southeastern Côte d’Ivoire where the ecological study of *Aedes* mosquitoes was carried out: **a** Ehania-V1 (covers the villages of Ehania-V1 and Akakro and represents the rural area without major and secondary paved roads. The site is close to the primary rainforest reserve). **b** Blockhauss (comprises the villages of Blockhauss and Petit-Cocody and represents the suburban area with only secondary paved roads. It is about 5 km away from the rainforest of Banco National Park). **c** Treichville (includes the sections of Jacques-Aka and Biafra and is the urban area with numerous major and secondary paved roads. It is located in the centre of Abidjan and is separated from Blockhauss by Ebrié lagoon)

The study was conducted in three settings in southeastern Côte d’Ivoire: Ehania-V1, Blockhauss and Treichville, representing rural, suburban and urban areas, respectively (Fig. 1). The village of Ehania-V1 (5°18′N, 3°04′W) belongs to the district of Aboisso some 140 km east of Abidjan. Ehania-V1 is a rural area with a population density of approximately 48 people/km^2^ and unpaved roads. The residencies are composed of traditional and ordinarily modern houses. This area is surrounded by industrial oil palm plantation (*Elaesis guineensis*) of 11,444 ha and 100 ha of preserved primary rainforest. The rainforest provides strong vegetation with dense canopy cover, trees with holes and bamboos and hosts non-human primates and birds.

Blockhauss (5°19′N, 4°00′W) is located within Abidjan bordered in its northern part by Banco National Park with over 3750 ha of rainforest. This setting is a suburban area with approximately 750 people/km^2^ and paved roads. The land use comprises a mixture of residential buildings, hospitals and schools. The residencies are ordinarily modern houses and some blocks with flats. Urbanization is underway in untapped spaces.

Treichville (5°18′N, 4°00′W) is situated in central Abidjan and separated from Blockhauss by the Ebrié Lagoon that has a width of approximately 4 km. This setting is an urban area with more than 1800 people/km^2^ and paved roads. The density of the population greatly increases during the daytime due to the convergence of people from other municipalities of Abidjan for trading, businesses and sports. The land use is essentially residential, commercial, cultural and sportive buildings, seaport, and public services such as schools and hospitals, filled with green spaces set apart. The residencies are mostly composed of blocks of flats and some ordinarily modern houses. Urbanization is completed due to the lack of availability of additional space for the construction of new houses.

In southeastern Côte d’Ivoire, the climate is warm and humid and overall, transitional from equatorial to tropical with four seasons. The seasons are more clearly distinguished by rainfall than temperature. The two rainy seasons are separated by a dry season. The main rainy season extends from May to July, while a short rainy season occurs in October and November. The main dry season extends from December to April. This classic sequence of seasonality has been disrupted due to strong climate variability [34]. The average annual precipitation ranges from 1200 to 2400 mm. The annual temperature is around 26.5 °C and the annual relative humidity (RH) ranges between 78 and 90 %.

### Design of sample collection

*Aedes* spp. eggs were sampled using the standard WHO ovitrap method [23, 24]. Ovitraps were metallic boxes cut with 400 cm^3^ volume and covered with black paint to attract gravid female *Aedes* mosquitoes in search of egg laying grounds. They were filled (75 % full) with distilled water mixed with rainwater and 10 % infusions of herbs (*Panicum maximum*) to increase the attractiveness of the ovitraps [35]. A 5 × 7 × 0.3 cm paddle made of hardboard, rough on one side and serving as oviposition substrate, was plunged in each box and left for a one-week period during each of six surveys. The rural area was divided into three ecozones: domestic, peridomestic and sylvatic. The suburban and urban areas were divided into domestic and peridomestic zones because there were no sylvatic zones. According to Cordellier et al. [23], domestic zone refers to the human-inhabited space, the peridomestic zone covers the vegetated environment surrounding the domestic zone in which humans usually discard artificial items that serve as breeding sites for *Aedes* mosquitoes, the sylvatic zone is primarily an undisturbed environment free of discarded containers that host forests with natural containers (e.g. tree holes) and wild animals. In our study, the peridomestic zone extended from the edge of the domestic zone to 300 m, while the sylvatic zone was located from 300 to 800 m around the village. During each of the six surveys, 50 ovitraps were repeatedly placed in the same location in each defined ecozone. In the human-inhabited zone, the ovitraps were suspended at 1.5 m above the ground to secure and protect them. In total, 900, 600 and 600 ovitraps were deployed in the rural, suburban and urban areas, respectively, from January 2013 to April 2014. In addition, from April to July 2013, we conducted socio-ecological investigations in 50 households representative of each study area in which ovitraps were placed to identify their sociogeographic status.

### Key socio-geographic characteristics

The socio-ecological investigations showed that the surveyed households varied highly along the increasing urbanization gradient. The mean number (mean ± standard error) of people per household was 5.9 ± 2.8 in the rural, 8.6 ± 2.1 in the suburban and 11.9 ± 3.2 in the urban areas. The potential resident containers were mainly discarded items (cans, pots, barrels, tyres) (*n* = 50; 58.0 %) and natural containers (coconut, tree hole, bamboo, snail shell) (46.0 %) in the rural area. The containers were mostly artificial and discarded receptacles with 78.0 and 92.0 % in the suburban and urban areas, respectively. The households stored water in the proportions of 88.0 % (*n* = 50) in the rural, 98.0 % in the suburban and 100 % in the urban areas.

### Sample laboratory treatment

In the field, the paddles, *Culex* egg rafts and remaining water from the ovitraps were separately stored in plastic boxes and then transported in a cold box to the laboratory. The paddles were dried during a period of 5 days at a temperature of 25 ± 1 °C, RH of 80–90 % and a 12:12 h light:dark photoperiod. They were screened with white, insect-proof, nylon netting to prevent eventual egg laying from other mosquitoes and potential predators. The paddles were then separately immersed in plastic cups (6 × 9 × 15 cm) 75 % filled with distilled water for attached egg hatching. The process was repeated three times after flooding of 5 days to maximize egg hatching. Emerging larvae were counted and recorded. As there was no reliable larval identification key, the larvae were reared until adult stage under identical conditions as paddle drying. To avoid overcrowding and limit mortality, at most 20 emerging larvae were placed per 200 ml plastic cup filled to 75 % with distilled water. Each plastic cup was labeled with a unique number of the sample, the study area and the date of collection. Larvae were fed each morning (07:00–08:00 h) with Tetra-Min Baby Fish Food®. Emerging pupae were transferred to new plastic cups using plastic pipettes for adult emergence. The cups containing the pupae were netted to avoid draining the newly hatched adults. *Culex* egg rafts were not dried but were stored at 15 ± 1 °C to avoid desiccation [36]. In addition, the larvae hatched from *Culex* egg rafts and the larvae of *Aedes*, *Culex* and *Eretmapodites* found in the collected water from the ovitraps were also separately reared until adult stage, under the same conditions as described above. During rearing, emerging adult *Aedes, Culex* and *Eretmapodites* specimens were identified to the species level using morphological criteria [23, 24, 37]. The mosquito individuals were stored at subgenus, species and sex levels and data were recorded in an entomology collection database.

### Statistical analysis

The measures of *Aedes* species proportions were expressed as the percentage of specimens among *Aedes* fauna for each study area and analysed using Fisher’s exact test to look at the relationship between the species composition and the study area and ecozone, and followed by the Proportion-test. Fisher’s exact test was used because expected numbers of specimens were equal or less than five. *Aedes* species richness was assessed as the number of collected species in each study area and compared using a one-way analysis of variance (ANOVA), followed by Bonferroni’s correction. The species diversity and dominance of *Aedes* spp. were estimated by Shannon-Weaver's index [38] and Simpson's index [39] and analysed by Kruskal-Wallis test because the log-transformed data exhibited significant deviations from normality. The abundance of *Aedes* spp. and *Ae. aegypti* was expressed as the mean number of specimens per ovitrap and analysed using repeated measures approaches in a generalized linear mixed model (GLMM) framework in order to take into account the possible interactions between the variables “month”, “study site” and “ecozone” [35]. To account for overdispersion due to excessive numbers of zeroes, the data were log-transformed [log (number of specimens + 1)] [36]. The log-transformed data were subjected to GLMM procedures and analysed as follows [35]. We compared the mean numbers of *Aedes* mosquito specimens per ovitrap between the study areas, the ecozones and the months using mixed-effects regression (*xtmixed* command), performed the joint tests of the interactions and the main effects of the study sites, the ecozones and months (*contrast* command) to understand the significant interactions, followed up the simple effects of each study area and ecozone over the months by pairwise comparisons (*margins* and *pwcompare* commands) and the *post-hoc* test of the trends (*contrast p.* operator) and the *post-hoc* test of the partial interaction (*contrast a.* operator). The mortality of the larvae during rearing was compared using negative binomial error. The extra sub-site, sylvatic zone, was excluded from the analysis when performing the comparisons between the study areas, and only included when the comparisons were conducted among the ecozones in the rural area. A significance level of 5 % was set for statistical testing. All data were analysed using Stata version 14.0 (Stata Corporation; College Station, TX, USA).

## Results

### Species composition of emerged adult mosquitoes

The mortality of the larvae hatched from *Aedes* spp. eggs during the rearing to adult stage was not statistically significant (all *P* > 0.05) thus making the comparison of emerged adults possible. Table 1 shows the species composition of adult *Aedes* spp. emerged from eggs collected from the different study areas. Totals of 2441, 2440 and 3098 adult *Aedes* spp. emerged from the eggs collected in the rural, suburban and urban areas, respectively. *Aedes* species belonged to three subgenera (*Stegomyia*, *Aedimorphus* and *Diceromyia*) in the rural areas, two subgenera (*Stegomiya* and *Aedimorphus*) in the suburban areas and a single subgenus (*Stegomyia*) in the urban areas. The species richness of *Aedes* spp. gradually decreased from the rural (eight species) to the suburban (three species) and urban (one species) areas. Fisher’s exact test indicated that *Aedes* species richness significantly varied from one study area to another (all *P* < 0.001). Proportion-testing indicated that there was a significant difference in *Aedes* species proportions in the rural (*χ*^2^ = 9411.15, *df* = 7, *P* < 0.0001) and the suburban (*χ*^2^ = 5052.86, *df* = 2, *P* < 0.0001) areas. *Aedes aegypti* was the predominant species with significantly higher proportions among *Aedes* fauna collected in the rural (*Z* = 18.91, *P* < 0.001) and suburban areas (*Z* = 7.83, *P* < 0.001), and the sole *Aedes* species in the urban areas. *Aedes africanus* and *Ae. dendrophilus* in the rural areas and *Ae. vittatus* in the suburban areas were found in significantly higher proportions. *Aedes metallicus* represented more than 1 % of the total *Aedes* fauna in the rural and the suburban areas whereas *Ae. furcifer*, *Ae. fraseri* and *Ae. luteocpehalus* were collected in lower proportions in the rural areas.

**Table 1:** Species composition of emerged adult *Aedes* spp. collected in the rural, suburban and urban areas of southeastern Côte d’Ivoire between January 2013 and April 2014

Subgenus	Species	Rural					Suburban					Urban				
		Female	Male	Total	%	MO ± SE	Female	Male	Total	%	MO ± SE	Female	Male	Total	%	MO ± SE
*Aedes* (*Stegomyia*)	*Aedes aegypti*	913	841	1754	63.3^a^	0.57 ± 0.05	1124	1035	2159	85.5^a^	1.20 ± 0.09	1521	1577	3098	100	1.97 ± 0.10
	*Aedes africanus*	137	139	276	9.4^b^	0.08 ± 0.02	0	0	0	0	0	0	0	0	0	0
	*Aedes dendrophilus*	122	139	261	8.0^b^	0.07 ± 0.02	0	0	0	0	0	0	0	0	0	0
	*Aedes metallicus*	22	14	36	1.3^c^	0.01 ± 0.01	20	12	32	1.2^c^	0.01 ± 0.01	0	0	0	0	0
	*Aedes usambara*	20	12	32	0.5^c^	0.01 ± 0.00	0	0	0	0	0	0	0	0	0	0
	*Aedes fraseri*	6	11	17	0.3^c^	0.01 ± 0.00	0	0	0	0	0	0	0	0	0	0
	*Aedes luteocephalus*	8	3	11	0.3^c^	0.00 ± 0.00	0	0	0	0	0	0	0	0	0	0
*Aedes* (*Aedimorphus*)	*Aedes vittatus*	0	0	0	0	0	130	119	249	6.5^b^	0.09 ± 0.02	0	0	0	0	0
*Aedes* (*Diceromyia*)	*Aedes furcifer*	20	14	34	0.7^c^	0.01 ± 0.01	0	0	0	0	0	0	0	0	0	0
Total	Abundance	1248	1173	2421	100	0.89 ± 0.06	1274	1166	2440	100	1.44 ± 0.09	1521	1577	3098	100	1.97 ± 0.10
	Richness (no. of spp.)	8					3					1				

Letters indicate the results of the Proportion-test. Groups that do not share the same letter for the same study area are significantly different (*P* < 0.05)

*Abbreviations*: *MO*, mean number per ovitrap; *SE*, standard error of the mean number per ovitrap

Non-*Aedes* mosquito species were also sampled in all study areas. Totals of 277, 108 and 67 specimens of *Culex* spp. were sampled from the rural, suburban and urban areas, respectively. In the rural area, *Culex* spp. was composed of three species, *Cx. nebulosus* (*n* = 277; 49.4 %), followed by *Cx. quinquefasciatus* (28.2 %) and *Cx. poicilipes* (22.4 %). The diversity of *Culex* spp. then decreased to a single species, *Cx. quinquefasciatus*, in the suburban (*n* = 108) and urban (*n* = 133) areas. *Eretmapodites* spp. was restricted to the rural area and composed of only one species, *Er. chrysogaster*, with 274 specimens.

### Richness, diversity and dominance of *Aedes* spp.

Table 2 presents the species richness, diversity and dominance of *Aedes* spp. in all of the study areas and different ecozones. *Aedes* spp. species richness was significantly different among the study areas (*F* = 18.60, *df* = 2, *P* = 0.0001) and ecozones (*F* = 9.24, *df* = 6, *P* < 0.0001), with higher numbers of species in the rural area and the peridomestic zone of the same area. The species diversity of *Aedes* spp. was statistically different among the study areas (*χ*^2^ = 14.00, *df* = 2, *P* = 0.0009) and ecozones (*χ*^2^ = 27.65, *df* = 6, *P* = 0.0001), with higher values for both diversity indices in the rural area and the sylvatic zone of the rural area. Moreover, *Aedes* spp. species dominance was significantly different among the study areas (*χ*^2^ = 13.86, *df* = 2, *P* = 0.0011) and ecozones (*χ*^2^ = 28.00, *df* = 6, *P* = 0.0001), with higher Simpson’s index values in the urban area and both peridomestic and domestic zones of the urban area.

**Table 2:** Species richness, diversity and dominance of *Aedes* spp. in the rural, suburban and urban areas and ecozones in southeastern Côte d’Ivoire

Area/ Ecozone	Richness	Shannon’s diversity index	Simpson’s dominance index
Area			
Rural	8^a^	1.39^a^	0.55^b^
Suburban	3^b^	0.57^a,b^	0.79^a,b^
Urban	1^b^	0^b^	1^a^
Ecozone			
Sylvatic^1^	5^a,b^	1.90^a^	0.28^c^
Peridomestic^1^	7^a,b^	1.23^a,b^	0.58^b,c^
Domestic^1^	5^b,c^	0.75^a,b,c^	0.77^a,b,c^
Peridomestic^2^	3^b,c^	0.67^a,b,c^	0.74^a,b,c^
Domestic^2^	3^b,c^	0.35^a,b,c^	0.89^a,b,c^
Peridomestic^3^	1^c^	0^c^	1^a^
Domestic^3^	1^c^	0^c^	1^a^

Letters indicate the results of one-way ANOVA test followed by Bonferroni correction (richness) and Kruskal-Wallis test (Shannon diversity index, Simpson dominance index). Groups that do not share the same letter are significantly different (*P* < 0.05)

^1^Ecozone in the rural area

^2^Ecozone in the suburban area

^3^Ecozone in the urban area

### Dynamics of *Aedes* spp. numbers

The highest mean numbers of emerged adult *Aedes* spp. were found in the urban setting (1.97 ± 0.10 *Aedes*/ovitrap/week), followed by the suburban (1.44 ± 0.09 *Aedes*/ovitrap/week) and rural (0.89 ± 0.06 *Aedes*/ovitrap/week) areas. The mean numbers of emerged adult *Aedes* spp. were significantly different between the rural and urban areas (*Z* = 5.01, *P *< 0.001). The effects and the interactions among the study areas, the ecozones and months, and the trends of *Aedes* spp. numbers over the months were statistically significant (Table 3).

**Table 3:** Effects, interactions and trends of *Aedes* spp. and *Ae. aegypti* numbers in the rural, suburban and urban areas in southeastern Côte d’Ivoire. The results are the outputs of the generalized linear mixed model (GLMM) procedures. The extra sub-site, sylvatic zone, was excluded from the data

		*Aedes* spp.			*Aedes aegypti*		
		*χ* ^2^	*df*	*P*	*χ* ^2^	*df*	*P*
1. Main effect & interaction							
1.1. Main effect							
Study area		20.16	2	< 0.00001*	50.37	2	< 0.00001*
Ecozone		43.76	1	< 0.00001*	26.32	1	< 0.00001*
Month		112.78	5	< 0.00001*	82.67	5	< 0.00001*
1.2. Interaction							
Study area × ecozone		7.09	2	0.0288*	13.25	2	0.0013*
Study area × month		15.90	10	0.1027	26.52	10	0.0031*
Ecozone × month		12.26	5	0.0314*	8.69	5	0.1221
Study area × ecozone × month		14.96	10	0.1335	8.29	10	0.6003
2. *Post-hoc* test of trends							
2.1. Study area	Trend						
Rural	Linear	2.55	1	0.1102	0.43	1	0.5109
	Quadratic	1.19	1	0.2752	2.81	1	0.0935
	Cubic	9.36	1	0.0022*	5.20	1	0.0225*
	Quartic	6.08	1	0.0136*	4.70	1	0.0302*
	Quintic	1.03	1	0.3099	0.06	1	0.7999
Suburban	Linear	7.31	1	0.0068*	4.86	1	0.275
	Quadratic	2.91	1	0.0880	0.92	1	0.3377
	Cubic	10.09	1	0.0015*	5.67	1	0.0173*
	Quartic	16.54	1	< 0.00001*	7.05	1	0.0079*
	Quintic	0.46	1	0.4969	3.58	1	0.0584
Urban	Linear	26.45	1	< 0.00001*	27.16	1	< 0.00001*
	Quadratic	0.02	1	0.8767	0.02	1	0.8798
	Cubic	15.22	1	0.0001*	15.64	1	0.0001*
	Quartic	28.58	1	< 0.00001*	29.42	1	< 0.00001*
	Quintic	2.67	1	0.1020	2.74	1	0.0981
Joint		128.68	15	< 0.00001*	109.47	1	< 0.00001*
2.2. Ecozone							
Peridomestic	Linear	23.32	1	< 0.00001*	21.11	1	< 0.00001*
	Quadratic	1.24	1	0.2658	0.53	1	0.4679
	Cubic	17.00	1	< 0.00001*	9.87	1	0.0017*
	Quartic	47.09	1	< 0.00001*	31.48	1	< 0.00001*
	Quintic	2.87	1	0.0900	0.21	1	0.6487
Domestic	Linear	8.28	1	0.0040*	3.96	1	0.0465*
	Quadratic	1.08	1	0.2978	1.71	1	0.1915
	Cubic	17.26	1	< 0.00001*	15.23	1	0.0001*
	Quartic	7.85	1	0.0051*	7.46	1	0.0063*
	Quintic	0.02	1	0.8846	0.23	1	0.6330
Joint		122.97	10	< 0.00001*	90.52	10	< 0.00001*

*Significant effects (*P* < 0.05)

*Abbreviations*: *χ* ^2^, chi-square; *df*, degrees of freedom; *P, P*-value

Table 4 summarises the geographical variation of adult *Aedes* species collected in each of the three study areas. In the rural areas, specimens of *Ae. africanus*, *Aedes dendrophilus*, *Aedes metallicus* and *Aedes fraseri* were collected in the domestic zone, while significant numbers of *Ae. aegypti* were sampled in the sylvatic zone. Emerged adult *Aedes* spp. mean numbers were significantly higher in the peridomestic zone with 1.36 ± 0.14 *Aedes*/ovitrap/week in the rural (Contrast = 0.50, *Z* = 5.16, *P *< 0.001), suburban (2.10 ± 0.15 *Aedes*/ovitrap/week; Contrast = -4.89, *Z* = -4.81, *P *< 0.001) and urban (2.80 ± 0.21 *Aedes*/ovitrap/week; Contrast = -0.49; *Z* = -4.85, *P *< 0.001) areas.

**Table 4:** Geographical variations in the number of emerged adult species of *Aedes* spp. in the rural, suburban and urban areas in southeastern Côte d’Ivoire

Species	Rural						Suburban				Urban			
	Sylvatic zone		Peridomestic zone		Domestic zone		Peridomestic zone		Domestic zone		Peridomestic zone		Domestic zone	
	*n*	MO ± SE	*n*	MO ± SE	*n*	MO ± SE	*n*	MO ± SE	*n*	MO ± SE	*n*	MO ± SE	*n*	MO ± SE
*Aedes aegypti*	132	0.15 ± 0.04	901	0,85 ± 0.12	721	0.84 ± 0.10	1353	1.64 ± 0.14	806	0.86 ± 0.11	1938	2.80 ± 0.21	1160	1.34 ± 0.16
*Aedes africanus*	106	0.10 ± 0.03	161	0.14 ± 0.04	9	0.01 ± 0.01	0	0	0	0	0	0	0	0
*Aedes dendrophilus*	121	0.11 ± 0.03	91	0.07 ± 0.03	49	0.05 ± 0.02	0	0	0	0	0	0	0	0
*Aedes metallicus*	2	0.00 ± 0.00	7	0.01 ± 0.00	27	0.02 ± 0.01	28	0.02 ± 0.01	4	0.01 ± 0.01	0	0	0	0
*Aedes usambara*	0	0	32	0.02 ± 0.02	0	0	0	0	0	0	0	0	0	0
*Aedes fraseri*	0	0	1	0.00 ± 0.00	16	0.01 ± 0.01	0	0	0	0	0	0	0	0
*Aedes luteocephalus*	0	0	11	0.01 ± 0.01	0	0	0	0	0	0	0	0	0	0
*Aedes vittatus*	0	0	0	0	0	0	201	0.15 ± 0.05	48	0.30 ± 0.00	0	0	0	0
*Aedes furcifer*	34	0.02 ± 0.02	0	0	0	0	0	0	0	0	0	0	0	0
Abundance	395	0.44 ± 0.03	1204	1.36 ± 0.14	822	1.01 ± 0.01	1582	2.10 ± 0.15	858	0.94 ± 0.11	1938	2.80 ± 0.21	1160	1.34 ± 0.16

*Abbreviations*: *n*, total number of specimens; *MO*, mean number of specimens per ovitrap per week; *SE*, standard error of the mean

**Fig. 2:**
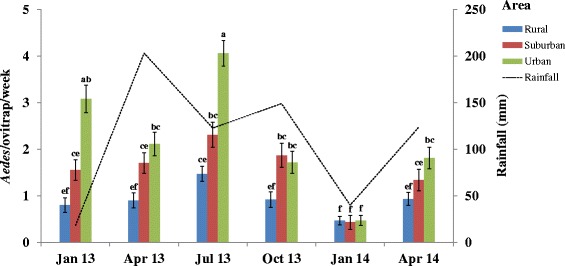
Monthly variations in mean numbers of emerged adult *Aedes* spp. as a function of the rainfall period. Rainfall was unexpectedly high in April 2013. The highest precipitations (374 mm) occurred in June 2013. Error bars show the standard error (SE) of the mean number of *Aedes* spp. per ovitrap. The letters indicate the results of the data analysed as repeated measures by generalized linear mixed model (GLMM) procedures. Groups that do not share a same letter are significantly different (*P* < 0.05)

Additional file 1: Table S1 indicates the seasonal variation of emerged adult *Aedes* spp. in all of the different study areas. *Aedes metallicus*, *Aedes usambara*, *Ae. fraseri*, *Ae. luteocephalus* and *Ae. furcifer* were not collected in January 2014 and April 2014. However, *Ae. aegypti* was sampled in all surveys in each study area. In all of the study areas, higher numbers of emerged adult *Aedes* spp. were found in July 2013 with 1.47 ± 0.18 *Aedes*/ovitrap/week in the rural, 2.31 ± 0.29 *Aedes*/ovitrap/week in the suburban and 4.06 ± 0.28 in the urban areas (Fig. 2). Conversely, the significantly respective lowest numbers of *Aedes* spp. were recorded in January 2014 with 0.47 ± 0.13 (all *P *< 0.05), 0.43 ± 0.17 (all *P *< 0.05) and 0.47 ± 0.11 (all *P *< 0.001) *Aedes*/ovitrap/week.

### Dynamics of *Ae. aegypti*

A total of 1754 (*n* = 2421; 63.3 ± 1.2 %) adult *Ae. aegypti* emerged from the eggs collected from the rural areas, 2159 (*n* = 2440; 85.5 ± 0.8 %) from the suburban and 3098 (*n* = 3098; 100 %) from the urban areas (Table 1). The highest mean numbers of *Ae. aegypti* were found in the urban setting, with 1.97 ± 0.10 *Ae. aegypti*/ovitrap/week). Considerably lower mean numbers were recorded in the rural and suburban settings, with 0.57 ± 0.05 and 1.20 ± 0.09 *Ae. aegypti*/ovitrap/week, respectively. *Aedes aegypti* mean numbers were significantly different between the urban and rural (*Z* = 6.23, *P* < 0.001), and the suburban and rural (*Z* = 2.15, *P* < 0.05) areas. The effects of, and interactions among the study areas, ecozones and months, and the trends of *Ae. aegypti* numbers over the months were statistically significant (Table 3).

**Fig. 3:**
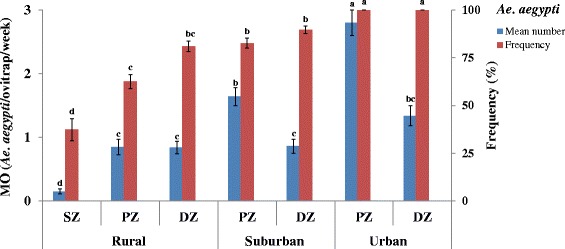
Geographical variations in mean numbers and frequencies of emerged adult *Ae. aegypti*. Error bars show the standard error (SE) of the mean number per ovitrap (MO) and the frequency. The letters indicate the results of the data analysed as repeated measures by Generalized Linear Mixed Model (GLMM) procedures for the mean number and Proportion-test for the frequency. Groups that do not share a same letter are significantly different (*P* < 0.05). *Abbreviations*: MO, mean numbers per ovitrap; SZ, sylvatic zone; PZ, peridomestic zone; DZ, domestic zone

Figure 3 shows the geographical variations of adult *Ae. aegypti* mean numbers and frequencies. Significantly higher mean numbers per ovitrap of *Ae. aegypti* were found in the peridomestic zones with 0.85 ± 0.12 *Ae. aegypti*/ovitrap/week in the rural (Contrast = 0.48, *Z* = 5.68, *P *< 0.001); 1.64 ± 0.14 *Ae. aegypti*/ovitrap/week in the suburban (Contrast = -0.36, *Z* = -3.65, *P *< 0.001); and 2.80 ± 0.21 *Ae. aegypti*/ovitrap/week in the urban (Contrast = -0.49, *Z* = -5.04, *P *< 0.001) settings. *Aedes aegypti* was collected in all of the ecozones of each study area. Its frequencies gradually increased from the sylvatic zone of the rural area (*n* = 395; 33.4 %) to the domestic zone of the urban area (*n* = 1160; 100 %) (*Z* = 31.43, *P *< 0.001).

**Fig. 4:**
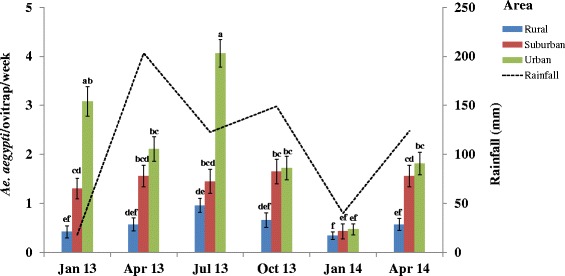
Monthly variations in mean numbers of emerged adult *Ae. aegypti* as a function of the rainfall period. Rainfall was unexpectedly high in April 2013. The highest precipitations (374 mm) occurred in June 2013. Error bars show the standard error (SE) of the mean number of *Ae. aegypti* per ovitrap. The letters indicate the results of the data analysed as repeated measures by generalized linear mixed model (GLMM) procedures. Groups that do not share a same letter are significantly different (*P* < 0.05)

Figure 4 shows the monthly variations of emerged adult *Ae. aegypti* mean numbers in relation to the rainfall. Emerged adult *Ae. aegypti* mean numbers significantly varied as a function of rainfall fluctuation in all study areas. The highest mean numbers were found during the rainy season in July 2013 with 0.96 ± 0.14 *Ae. aegypti*/ovitrap/week in the rural and 4.06 ± 0.28 *Ae. aegypti*/ovitrap/week in the urban areas, and in October 2013 with 1.65 ± 0.25 *Ae. aegypti*/ovitrap/week in the suburban areas. In urban areas, *Ae. aegypti* mean numbers dramatically declined in January 2014 (0.47 ± 0.11 *Ae. aegypti*/ovitrap/week) compared to July 2013 (Contrast = -1.25, *Z* = -7.88, *P *< 0.001). In the same study area, *Ae. aegypti* numbers were significantly higher in January 2013 (3.08 ± 0.11 *Ae. aegypti*/ovitrap/week) compared to January 2014 (Contrast = -1.02, *Z* = -6.57, *P *< 0.001).

## Discussion

To our knowledge, this is the first study exploring oviposition ecology of *Aedes* mosquitoes in variously urbanized settings of Côte d’Ivoire. Importantly, several species of *Aedes* were involved in previous dengue and yellow fever outbreaks in Côte d’Ivoire [18–22]. The outbreaks might be explained by the paucity of vector control strategies. A deeper understanding of the modifications induced by urbanization in the ecology of *Aedes* mosquitoes is crucial. Our data suggest that *Aedes* oviposition ecology and species composition, and *Ae. aegypti* dynamics differ from rural to suburban and urban areas in southeastern Côte d’Ivoire.

Our data highlighted that the mean numbers of emerged adult *Aedes* spp. increase from rural to urban areas. An increase in *Aedes* species prevalence and abundance by urbanization was indicated by Li et al. [2]. This phenomenon is probably due to elevated numbers of *Aedes* breeding sites such as tyres, discarded cans or water storage containers, provided by urbanizing environment [2]. In addition, an urbanized environment accelerates *Aedes* mosquito development and survivorship [2]. By increasing *Aedes* spp. abundance, urbanization could potentially aggravate epidemic risk factors for arbovirus.

Our results showed that urbanization alters *Aedes* mosquito species composition towards dominance of *Ae. aegypti* in the urban areas, while rural and suburban areas favour other wild *Aedes* species, including *Ae. vittatus*, *Ae. dendrophilus*, *Ae. africanus*, *Ae. luteocephalus*, *Ae. furcifer*, *Ae. metallicus*, *Ae. usambara* and *Ae. fraseri. Aedes aegypti* eggs are expected to be more desiccation-resistant [23, 24]; this might raise their ability to survive in a deforested environment such as the urban areas exposed to direct sunlight and thus increase the species geographical invasion. Conversely, the wild *Aedes* species collected only in the rural and suburban settings probably originated from a natural environment such as the preserved rainforest and the Banco National Park forest, respectively. The disappearance of wild *Aedes* species in the urban settings might be explained by the destruction of the natural environment for building houses and other infrastructure. The removal of vegetation due to house constructions and other infrastructure developments results in direct exposure of *Aedes* spp. breeding sites to solar radiation. The wild *Aedes* species eggs from rural settings could be protected against solar radiation by rainforest canopy [40] since they are laid in tree holes [41] and bamboo internodes [42] filled by rainwater and maintained under low temperature. It is conceivable that wild *Aedes* species that lay more fragile and desiccation-sensitive eggs remain confined to the rural areas, mainly in the rainy forest [23, 24]. Additional field manipulations and experiments under controlled laboratory conditions testing the different *Aedes* species egg desiccation-resistance levels may be useful to better understand the segregation among the species and the population growth rates. Indeed, the forest-dwelling *Aedes* species that are still present in the rural areas may play a key role as bridge vectors between the sylvatic cycles of dengue, yellow fever and other viruses among non-human primates and humans [17]. The vector role of these *Aedes* species is subtle and difficult to trace, and often remains undetected because there are no traditional epidemiological risk indicators such as the house index, container index or Breteau index [43]. However, the exclusive existence of predators such as *Er. chrysogaster* in the rural areas might influence the abundance of *Aedes* species [27, 42]. *Er. chrysogaster* is also suspected to transmit arboviruses in tropical Africa [23]. In summary, the segregation induced by urbanization in *Aedes* species diversity is consistent with the known arbovirus transmission cycles in tropical Africa [17] and merits further consideration for dengue and yellow fever surveillance.

Our results suggested that the geographical and seasonal variations of *Aedes* spp. are associated with urbanized settings. The preference of *Aedes* spp. to lay eggs in the peridomestic vicinity confirms previous findings from urban areas in Brazil [44] and Vietnam [45]. Peridomestic premises are in close proximity to human residencies, and hence the principal blood-meal sources of adult *Aedes* mosquitoes. Furthermore, they also provide ideal ecosystems such as dense vegetation favourable for *Aedes* spp. refugia [46] and natural breeding sites such as tree holes [39, 42, 47] and artificial containers as discarded cans and old vehicle tyres [48, 49]. Regarding the seasonal variation, *Aedes* spp. mean numbers were strongly associated with rainfall patterns, history, variability and intensity. The fluctuations in *Aedes* spp. counts could be influenced by seasonal flooding-drying cycles as reported in Côte d’Ivoire [18] and Brazil [44]. *Aedes* spp. eggs probably enter into a dormant stage to withstand desiccation periods during the dry season, while precipitations might flood the breeding sites and increase the abundance of *Aedes* spp. [50]. However, the sudden decline of *Aedes* spp. numbers in October 2013 in the urban setting might be due to heavy precipitations and exacerbated flushing of their eggs because of the lack of protective vegetation in the built-up environment [51].

Finally, our findings revealed that *Ae. aegypti* is the most common species along the increasing urbanization gradient and the unique *Aedes* species in urban settings, thus suggesting particular attention on its egg laying patterns and population dynamics. *Aedes aegypti* is an urban species that preferentially feeds on humans [52] and is well adapted to live in close proximity to human habitats [53]. Such highly anthropophilic behaviour may enhance human-to-human transmission of arboviruses and trigger dengue and yellow fever outbreaks. The dominance of *Ae. aegypti* in still urbanizing and already urbanized areas of Africa is well documented [8, 9, 18] and is possibly due to its plastic oviposition behaviour allowing the colonization of natural and artificial environments [9, 42]. Otherwise, the rising occurrence of *Ae. aegypti* was also coupled with the increasing presence of another urban, anthropophilic and sympatric species, *Cx. quinquefasciatus* [54–56] and the lack of predators such as *Er. chrysogaster* [27] in the urban area. In contrast, the specimens of *Ae. aegypti* unexpectedly collected in the sylvatic zone are, perhaps, members of the *Aedes aegypti formosus*, the ancestral progenitor of *Aedes aegypti aegypti* and the only sylvan form known in West Africa [30, 31]. *Aedes aegypti formosus* has no white scales on the first abdominal tergite and a dark or black cuticle. This subspecies is exophilic, preferentially feeds on wild animals and breeds in natural containers such as tree holes [32, 57, 58], whereas, *Ae. aegypti aegypti* has scales on the first abdominal tergite and a lightly tanned cuticle and tends to be endo- and anthropophilic and breed in man-made containers [31]. However, contrary to East Africa [29, 30, 57], the scaling and behavioural patterns do not match with the discrete genetic differences in allozymes and microsatellites for *Ae. aegypti* collected in West Africa [13, 31, 33, 40, 59]. This results in confusion over morphological distinction between the two forms [13]. Due to these ambiguities, we were not able to confirm which *Ae. aegypti* form was represented among the sylvan specimens collected in the rural area. Above all, the urban and sylvan forms of *Ae. aegypti* are both competent arbovirus vectors in West Africa [13].

Urbanization continues at a rapid pace in Côte d’Ivoire, particularly in the southeastern part resulting in drastic segregation among *Aedes* species by favouring *Ae. aegypti* and restricting wild *Aedes* species to rural areas. These trends were paralleled by recurrent resurgences of yellow fever and dengue in recent years. However, yellow fever is historically well known as a key factor having forced the transfer of the colonial capital of Côte d’Ivoire from Grand-Bassam to Abidjan in 1899 [60]. Despite this historical and present background, the resurgence of yellow fever and dengue outbreaks is not resolved and their sporadic occurrence creates major public health concerns [60]. Between 2001 and 2007, 1468 suspected, 41 confirmed and 26 fatal cases of yellow fever were reported. During the period of 2007–2001, 111 suspected with 31 confirmed and 43 deadly cases of yellow fever were notified. The incidence of yellow fever gradually increased and peaked in 2011 with 79 cases and 35 deaths. In 2008, nine cases of yellow fever and two cases of dengue DENV-3 were recorded. In 2010, 13 confirmed and two fatal cases of yellow fever, and one deadly case of DENV-1 were reported. The strengthened warning systems and the operated vector control are usually performed in urban areas, mainly in Abidjan. Our study suggests that while vector control should focus on urban areas, rural areas are important as they may serve as transition zones for (re-)introduction of arboviral diseases through sylvatic bridge vectors. Because rural areas host various wild vectors, they act as a potential reservoir and originator of arboviruses from which urban areas are (re-)infected. Therefore, rural areas also need to be considered when elaborating and applying arbovirus vector surveillance and control strategies. *Aedes* species control strategies could apply the lethal ovitrap [61] and autocidal [62] gravid ovitrap-based on mass trapping method.

## Conclusions

In arbovirus foci of the southeastern Côte d’Ivoire, urbanized environment correlates with a substantially higher abundance in *Aedes* species and a regression of the *Aedes* wild species towards a unique presence of *Ae. aegypti. Aedes aegypti* is expected to drive arbovirus transmission in the urban areas, while other species probably serve as potential bridge vectors between sylvatic and urban cycles of human arboviral infections in the rural areas. Our findings provide valuable information on *Aedes* spp. ecology patterns in variously urbanized settings and therefore suggest that the rural areas also need to be considered when implementing arbovirus vector surveillance and control strategies.

## Additional file

Additional file 1: Table S1. Seasonal variations in the number of emerged adult species of *Aedes* spp. in the rural, suburban and urban areas in southeastern Côte d’Ivoire. (DOCX 20 kb)

## Abbreviations

DZ: Domestic zone
GLMM: Generalized linear mixed model
MO: Mean number per ovitrap
PZ: Peridomestic zone
SZ: Sylvatic zone
