# Supplementary material for: Oviposition ecology and species composition of Aedes spp. and Aedes aegypti dynamics in variously urbanized settings in arbovirus foci in southeastern Côte d’Ivoire
Source: Parasit Vectors. 2016 Sep 29;9:523. doi: 10.1186/s13071-016-1778-9 (PMC5041276; doi:10.1186/s13071-016-1778-9)
Supplement: Additional file 1: Table S1. — Seasonal variations in the number of emerged adult species of Aedes spp. in the rural, suburban and urban areas in southeastern Côte d’Ivoire. (DOCX 20 kb) [file 13071_2016_1778_MOESM1_ESM.docx]

**Additional file 1: Table S1.** Seasonal variations in the number of emerged adult species of *Aedes* spp. in the rural, suburban and urban areas in southeastern Côte d’Ivoire

| **Species** | **Rural** | | | | | | **Suburban** | | | | | | **Urban** | | | | | |
| --- | --- | --- | --- | --- | --- | --- | --- | --- | --- | --- | --- | --- | --- | --- | --- | --- | --- | --- |
|  | **Jan-Apr 2013** | | **Jul-Oct 2013** | | **Jan-Apr 2014** | | **Jan-Apr 2013** | | **Jul-Oct 2013** | | **Jan-Apr 2014** | | **Jan-Apr 2013** | | **Jul-Oct 2013** | | **Jan-Apr 2014** | |
|  | ***n*** | **MO ± SE** | ***n*** | **MO ± SE** | ***n*** | **MO ± SE** | ***n*** | **MO ± SE** | ***n*** | **MO ± SE** | ***n*** | **MO ± SE** | ***n*** | **MO ± SE** | ***n*** | **MO ± SE** | ***n*** | **MO ± SE** |
| *Aedes aegypti* | 603 | 0.50 ± 0.09 | 759 | 0.80 ± 0.10 | 392 | 0.45 ± 0.07 | 778 | 1.43 ± 0.15 | 843 | 1.55 ± 0.18 | 538 | 0.75 ± 0.13 | 1,308 | 2.55 ± 0.02 | 1,210 | 2.70 ± 0.19 | 580 | 1.03 ± 0.13 |
| *Aedes africanus* | 94 | 0.08 ± 0.03 | 105 | 0.09 ± 0.03 | 77 | 0.07 ± 0.03 | 0 | 0 | 0 | 0 | 0 | 0 | 0 | 0 | 0 | 0 | 0 | 0 |
| *Aedes dendrophilus* | 84 | 0.06 ± 0.03 | 79 | 0.08 ± 0.03 | 98 | 0.08 ± 0.03 | 0 | 0 | 0 | 0 | 0 | 0 | 0 | 0 | 0 | 0 | 0 | 0 |
| *Aedes metallicus* | 33 | 0.02 ± 0.02 | 3 | 0.00 ± 0.00 | 0 | 0 | 13 | 0.02 ± 0.02 | 0 | 0 | 19 | 0.02 ± 0.02 | 0 | 0 | 0 | 0 | 0 | 0 |
| *Aedes usambara* | 32 | 0.02 ± 0.02 | 0 | 0 | 0 | 0 | 0 | 0 | 0 | 0 | 0 | 0 | 0 | 0 | 0 | 0 | 0 | 0 |
| *Aedes fraseri* | 16 | 0.01 ± 0.01 | 1 | 0.00 ± 0.00 | 0 | 0 | 0 | 0 | 0 | 0 | 0 | 0 | 0 | 0 | 0 | 0 | 0 | 0 |
| *Aedes luteocephalus* | 0 | 0 | 11 | 0.01 ± 0.01 | 0 | 0 | 0 | 0 | 0 | 0 | 0 | 0 | 0 | 0 | 0 | 0 | 0 | 0 |
| *Aedes vittatus* | 0 | 0 | 0 | 0 | 0 | 0 | 55 | 0.06 ± 0.03 | 166 | 0.21 ± 0.06 | 28 | 0.03 ± 0.03 | 0 | 0 | 0 | 0 | 0 | 0 |
| *Aedes furcifer* | 31 | 0.02 ± 0.01 | 3 | 0.00 ± 0.00 | 0 | 0 | 0 | 0 | 0 | 0 | 0 | 0 | 0 | 0 | 0 | 0 | 0 | 0 |
| Abundance | 893 | 0.85 ± 0.11 | 961 | 1.17 ± 0.12 | 567 | 0.69 ± 0.09 | 846 | 1.62 ± 0.15 | 1,009 | 2.08 ± 0.19 | 585 | 0.83 ± 0.14 | 1,308 | 2.55 ± 0.02 | 1,210 | 2.70 ± 0.19 | 580 | 1.03 ± 0.13 |
| *Abbreviations*: *n*, number of specimens; MO, mean number of specimens per ovitrap per week; SE, standard error of the mean | | | | | | | | | | | | | | | | | | |
